# Dissecting strategies to tune the therapeutic potential of SARS-CoV-2–specific monoclonal antibody CR3022

**DOI:** 10.1172/jci.insight.143129

**Published:** 2021-01-11

**Authors:** Caroline Atyeo, Matthew D. Slein, Stephanie Fischinger, John Burke, Alexandra Schäfer, Sarah R. Leist, Natalia A. Kuzmina, Chad Mire, Anna Honko, Rebecca Johnson, Nadia Storm, Matthew Bernett, Pei Tong, Teng Zuo, Junrui Lin, Adam Zuiani, Caitlyn Linde, Todd Suscovich, Duane R. Wesemann, Anthony Griffiths, John R. Desjarlais, Boris D. Juelg, Jaap Goudsmit, Alexander Bukreyev, Ralph Baric, Galit Alter

**Affiliations:** 1 Ragon Institute of MGH, MIT, and Harvard, Cambridge, Massachusetts, USA.; 2 Program in Virology, Division of Medical Sciences, Harvard University, Boston, Massachusetts, USA.; 3 Program in Immunology and Virology, University of Duisburg-Essen, Essen, Germany.; 4 Department of Epidemiology, Gillings School of Global Public Health, University of North Carolina at Chapel Hill, Chapel Hill, North Carolina, USA.; 5 Department of Pathology, University of Texas Medical Branch, Galveston, Texas, USA.; 6 Galveston National Laboratory, Institute for Human Infections and Immunity, University of Texas Medical Branch, Galveston, Texas, USA.; 7 Department of Microbiology, Boston University School of Medicine, Boston, Massachusetts, USA.; 8 National Emerging Infectious Diseases Laboratories, Boston University, Boston, Massachusetts, USA.; 9 Xencor, Monrovia, California, USA.; 10 Department of Medicine, Brigham and Women’s Hospital; Division of Allergy and Clinical Immunology; and Division of Genetics, Harvard Medical School, Boston, Massachusetts, USA.; 11 SeromYx Systems, Cambridge, Massachusetts, USA.; 12 Departments of Epidemiology and Immunology and Infectious Diseases, Harvard T.H. Chan School of Public Health, Boston, Massachusetts, USA.; 13 Department of Microbiology & Immunology, University of Texas Medical Branch, Galveston, Texas, USA.; 14 Departments of Microbiology and Immunology and Genetics, School of Medicine, and; 15 Lineberger Comprehensive Cancer Center, University of North Carolina, Chapel Hill, North Carolina, USA.

**Keywords:** COVID-19, Immunology, Antigen, Epidemiology

## Abstract

The rapid spread of severe acute respiratory syndrome coronavirus 2 (SARS-CoV-2), coupled with a lack of therapeutics, has paralyzed the globe. Although significant effort has been invested in identifying antibodies that block infection, the ability of antibodies to target infected cells through Fc interactions may be vital to eliminate the virus. To explore the role of Fc activity in SARS-CoV-2 immunity, the functional potential of a cross–SARS-reactive antibody, CR3022, was assessed. CR3022 was able to broadly drive antibody effector functions, providing critical immune clearance at entry and upon egress. Using selectively engineered Fc variants, no protection was observed after administration of WT IgG1 in mice or hamsters. Conversely, the functionally enhanced Fc variant resulted in increased pathology in both the mouse and hamster models, causing weight loss in mice and enhanced viral replication and weight loss in the more susceptible hamster model, highlighting the pathological functions of Fc-enhancing mutations. These data point to the critical need for strategic Fc engineering for the treatment of SARS-CoV-2 infection.

## Introduction

The recent pandemic of coronavirus disease 2019 (COVID-19), caused by the severe acute respiratory syndrome coronavirus 2 (SARS-CoV-2), has resulted in millions of infections and more than 1 million deaths globally in a remarkably short period (1). Although most human coronavirus infections cause mild respiratory disease, SARS-CoV and Middle East respiratory syndrome coronavirus (MERS-CoV) resulted in fatality rates of 10% and 36%, respectively (2, 3). Although the precise death counts remain unclear for SARS-CoV-2, fatality rates appear to be lower than those of SARS-CoV and MERS (4). But its alarming rates of spread, linked to transmission during the asymptomatic stage of infection, render this pathogen particularly lethal. Although the development of vaccines against SARS-CoV-2 are underway, therapeutics are urgently needed to support those with more severe infection. Among the therapeutics, several antivirals and antiinflammatories are under investigation (5, 6). In addition, anti–SARS-CoV-2 monoclonal therapeutics also have been proposed to control and clear the infection.

The coronavirus spike protein (S), found on the surface of coronaviruses, is involved in viral attachment and fusion (7). Treatment with monoclonal antibodies against SARS-CoV S protein has been shown to protect mice from viral pathogenesis (8). Delivery of both neutralizing and non-neutralizing antibodies against the MERS virus afforded protection (9–11), highlighting the potential importance both of blockade of infection and targeted immune-mediated clearance of the virus/virally infected cells in protection from infection. Likewise, both neutralization and antibody-dependent cellular cytotoxicity (ADCC) have been linked to protection in SARS-infected individuals (12) and animal models (13). Given the remarkable infectiousness of SARS-CoV-2, with an estimated R_0_ approximately 2.5 (14–18), strategies to provide complete protection from infection may require both blocking and postinfection eliminating-antibody functions for maximal immunity. However, data from SARS-CoV–immunized nonhuman primates pointed to the potential role of neutralizing antibodies in enhancing disease, via the induction of inflammatory responses (19), suggesting that caution is warranted in the application of monoclonal antibody therapeutics for SARS-CoV-2 treatment.

To begin to explore the potential immune-protective versus immunopathological role of antibodies, we focused on an antibody derived from a SARS-CoV–infected individual, CR3022, that targets a conserved epitope of the receptor binding domain (RBD) and that binds to the SARS-CoV-2 RBD (20). Because this cross-reactive antibody exhibits limited neutralization, despite binding to conserved determinants on the RBD (21), the antibody offered an opportunity to explore Fc-dependent effects on SARS-CoV-2 not confounded by neutralization. Moreover, given that CR3022 continues to bind to RBD even in the presence of angiotensin-converting enzyme 2 (ACE2) (20–22), CR3022 has the potential to confer eradication of infected cells even in the setting of high ACE2 secretion (23). Thus, here we coupled Fc functional profiling, Fc engineering, and in vivo profiling to examine the role of Fc effector function on the response to SARS-CoV-2 infection. Distinct Fc functional profiles resulted in enhancement of disease, pointing to antibody mechanisms of action that may be detrimental when developing antibody therapeutics against the virus.

## Results

### CR3022 drives innate immune activity against SARS-CoV-2.

Although great effort is underway to identify potent neutralizing antibodies against SARS-CoV-2, it remains uncertain whether neutralization alone, particularly in the upper respiratory tract, will be sufficient to provide complete protection against this highly infectious pathogen. Instead, past studies in MERS infection suggest that additional antibody functions, beyond neutralization, track with protective immunity (13). Thus, the ability to clear virus or infected cells that escape restriction in the upper respiratory tract may be essential to fully prevent disease. Here we focused on a first-in-class monoclonal antibody, initially cloned from a SARS-CoV–infected individual, because of its ability to bind to a mutated neutralization-resistant form of the SARS-CoV S1-RBD (22). CR3022 binds to both SARS-CoV and SARS-CoV-2, with a binding footprint that likely provides the antibody with the ability to bind broadly across SARS-CoV mutants, such as SARS-CoV-2, and to continue to bind in the setting of ACE2 binding to the RBD (20, 21). However, whether this antibody was able to drive additional functions of potential therapeutic utility remained unclear.

**Figure 1:**
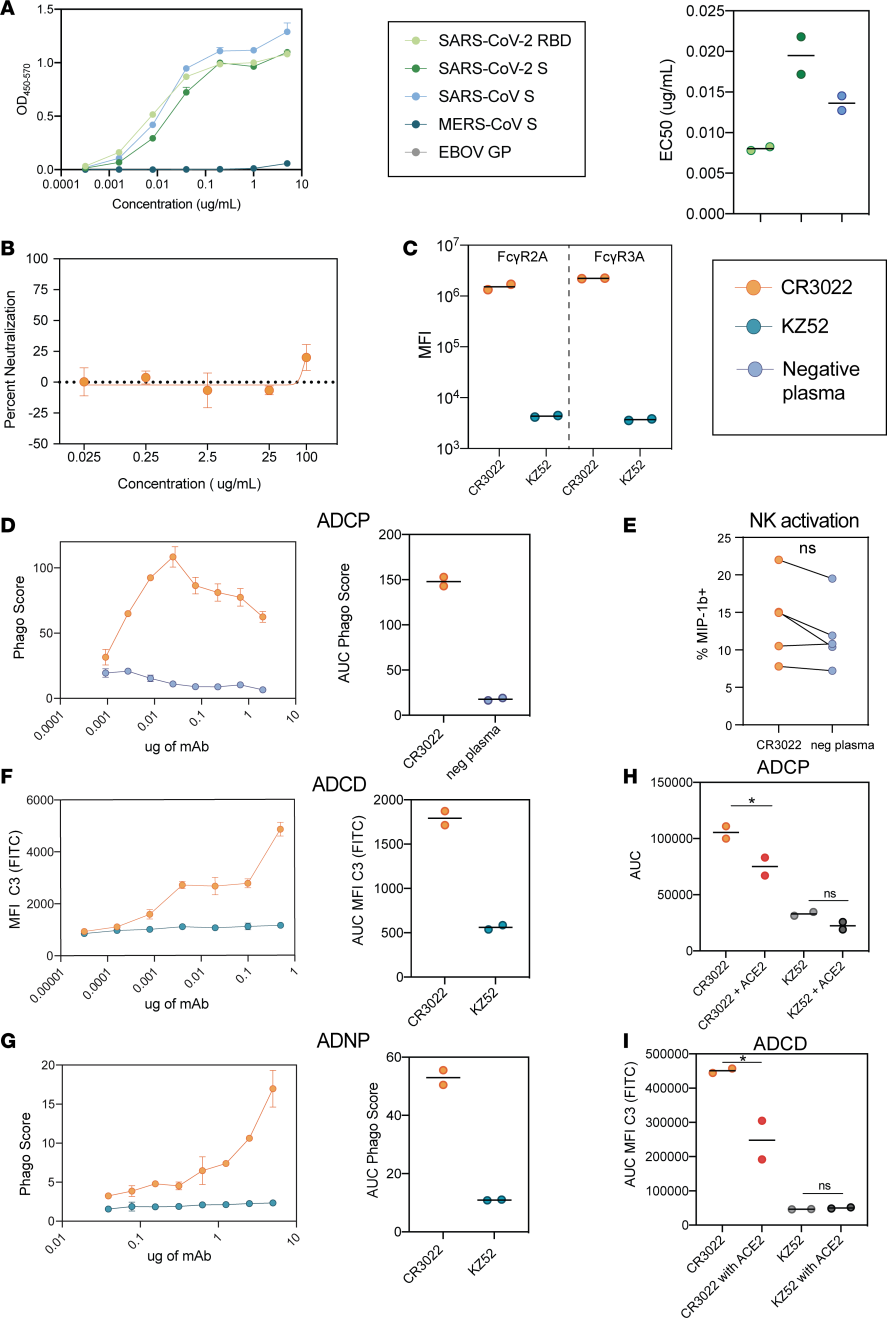
CR3022 drives effector function against SARS-CoV-2. (**A**) CR3022 was serially diluted and tested for its ability to bind the spike protein of different coronaviruses by ELISA (left). Data are represented as the OD_450_ values background subtracted from the reference OD_570_ value. Each dot represents the average of 2 replicates. The bar plot displays the EC_50_ for each antigen. MERS RBD and Ebola virus (EBOV) glycoprotein are not displayed because their respective EC_50_ was each infinite. The bars represent the average of 2 replicates, and the error bars represent the standard deviation. (**B**) CR3022 was serially diluted and preincubated with SARS-CoV-2 before adding to the virus and antibody to Vero E6 cells. Percentage neutralization was determined by the percentage reduction in plaque counts compared with a vehicle control. Data represent the means of triplicates and error bars represent the standard deviation. (**C**) CR3022 and a control EBOV-specific antibody binding to FcγR2a and 3a were evaluated using Luminex with a serial dilution of CR3022. The AUC was calculated for the MFI values. The bar represents the mean of 2 replicates. (**D**–**G**) CR3022 was evaluated for its ability to drive ADCP (**D**), NK activity (as measured by MIP-1b activity) (**E**), ADCD (**F**), and ADNP (**G**). For the line graphs (left), each dot represents the mean of 2 replicates. For the bar graphs, values are represented as the mean AUC of 8 serial dilutions run in replicate. The error bars for the dots and the AUC represent the standard deviation. For the before-after plot (**E**), each dot represents the activity of 1 donor after incubation with CR3022 or serum. Significance for NK activation (**E**) was determined by a Wilcoxon matched pairs signed rank test, *P < 0.05. (**H** and **I**) The ability of CR3022 and a control EBOV-specific antibody (KZ52) to drive ADCP (**H**) and ADCD (**I**) in the presence of ACE2 was analyzed. The bars indicate the average AUC of 8 serial dilutions run in 2 replicates. The error bars represent the standard deviation. Significance was determined by a 1-way ANOVA test followed by Tukey’s multiple-comparison test. *P < 0.05.

To begin, we confirmed the ability of CR3022 to bind to SARS-CoV and SARS-CoV-2 S as well as SARS-CoV-2 RBD and related CoV spike proteins (Figure 1A). As expected, CR3022 bound tightly to the SARS-CoV S, against which it was cloned. The antibody also bound SARS-CoV-2 RBD and S. In contrast, CR3022 did not bind to the MERS S, highlighting the specificity of this antibody for SARS-related viruses. Although it was able to bind to SARS-CoV-2 RBD and potently neutralize SARS-CoV in vitro (22), limited authentic SARS-CoV-2 neutralization was observed even at very high antibody concentrations (Figure 1B) as has been previously observed (21).

Emerging data point to significant differences across monoclonal antibodies in their ability to drive Fc effector functions (24). Both the stoichiometry and geometry of binding have been implicated in modulating antibody effector function (25). Given the peculiar angle with which CR3022 interacts with the SARS-CoV-2 RBD (21), we next examined the ability of CR3022 to drive Fc effector function. CR3022 bound to SARS-CoV-2 S was able to bind to Fc gamma receptors 2a and 3a (FcγR2a and FcγR3a) (Figure 1C), whereas no FcR binding was observed with the control EBOV-specific antibody (KZ52) bound to SARS-CoV-2 S, likely due to lack of EBOV monoclonal binding to the SARS-CoV-2 antigen (25). Along the same lines, CR3022 was able to drive antibody-dependent cellular (monocyte) phagocytosis (ADCP), antibody-dependent NK cell activation (ADNKA, as measured by macrophage inflammatory protein 1b [MIP-1b] expression), antibody-dependent complement deposition (ADCD), and antibody-dependent neutrophil phagocytosis (ADNP) (Figure 1, D–G). Moreover, CR3022 was still able to drive antibody effector function, namely ADCP and ADCD, in the presence of ACE2 (Figure 1, H and I). Thus, unlike neutralizing antibodies, many of which compete with ACE2 binding, CR3022 may still drive antibody-effector clearance of virus or infected cells even upon ACE2 upregulation following infection (26).

### Comparison of CR3022 and other SARS-CoV-2 monoclonals.

**Figure 2:**
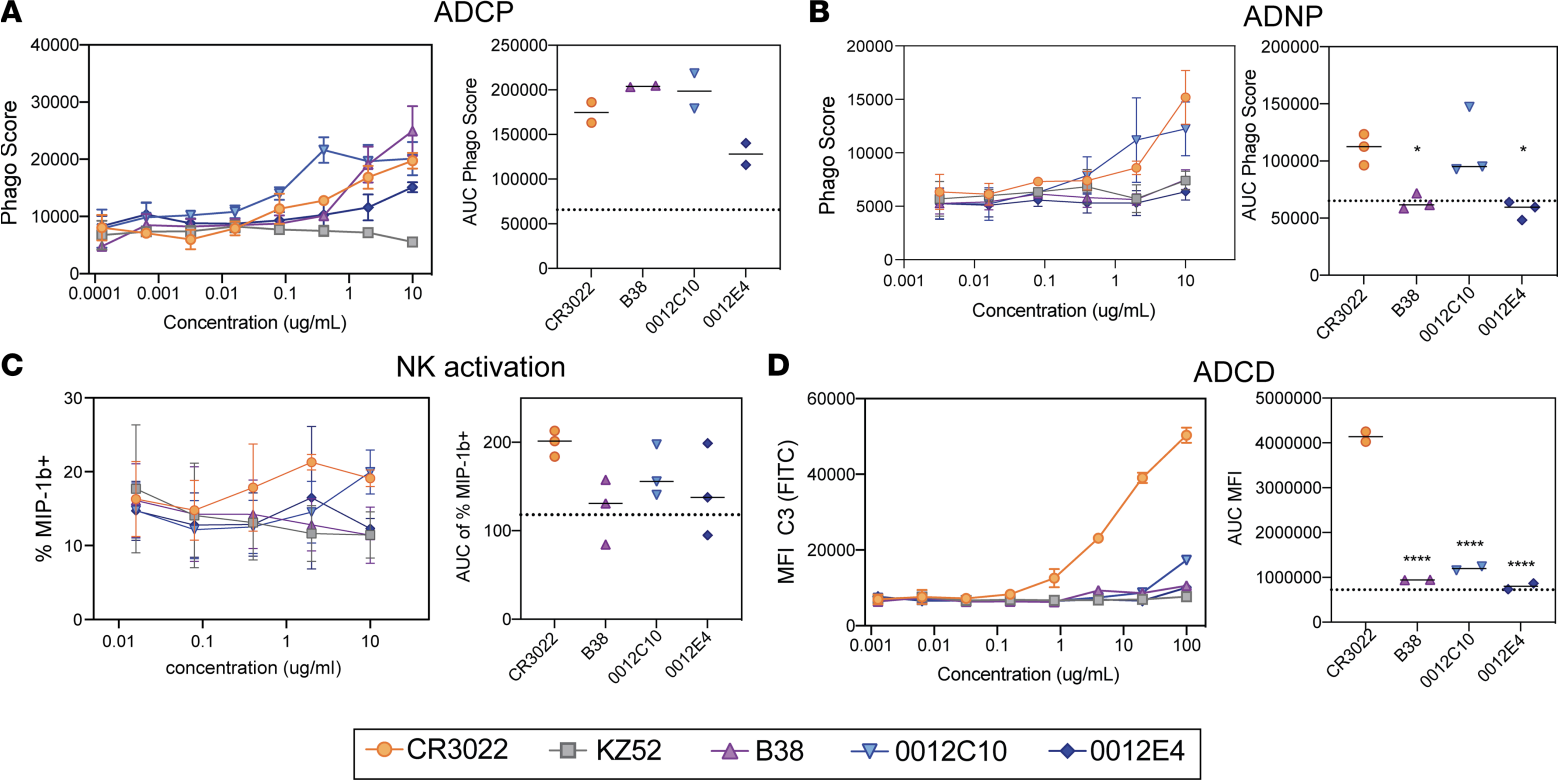
CR3022 possesses comparable Fc activity to neutralizing and non-neutralizing SARS-CoV-2 monoclonal antibodies. (**A**–**D**) CR3022 was tested for its ability to drive ADCP (**A**), ADNP (**B**), NK cell activation (**C**), or ADCD (**D**) compared with other SARS-CoV-2 RBD-targeting monoclonals. For the line graphs, each dot represents the mean of 2 replicates. For NK cell activation, 3 donors were used. For the bar graphs, the values are represented as the AUC of serial dilutions. The dotted line represents the average AUC value for the control EBOV-targeting antibody (KZ52). The error bars represent the standard deviation between the replicates. Significance was determined by an ordinary 1-way ANOVA followed by Tukey’s multiple-comparison test. *P < 0.05, ****P < 0.0001.

To fully probe the therapeutic potential of CR3022, we compared the functional activity of CR3022 with recently published (27) or discovered monoclonal antibodies specifically cloned from SARS-CoV-2–infected individuals. Specifically, the ability of CR3022 to mediate ADCP, NK activation, ADCD, and ADNP was compared with 2 neutralizing antibodies (B38 and 0012C10) and 1 non-neutralizing antibody (0012E4) (Table 1). In addition, we used an EBOV-targeting antibody (KZ52) as a negative control. All SARS-CoV-2 RBD-targeting antibodies drove similar levels of ADCP and ADNP (Figure 2, A and B), with CR3022 comparable to all other SARS-CoV-2 monoclonals. Conversely, CR3022 drove slightly more NK activation, as represented by MIP-1b secretion (Figure 2C), and significantly more complement deposition (ADCD) compared with the other 3 SARS-CoV-2 RBD-targeting antibodies (Figure 2D). Thus, CR3022 exhibited similar if not superior antibody-effector function compared with neutralizing and other non-neutralizing antibodies. Moreover, given the emerging role for ADCD in providing vaccine-mediated protection in vivo (28), the ability of CR3022 to facilitate ADCD makes CR3022 an ideal candidate for potential therapeutic applications.

### Fc engineering can tune CR3022 effector function.

**Figure 3:**
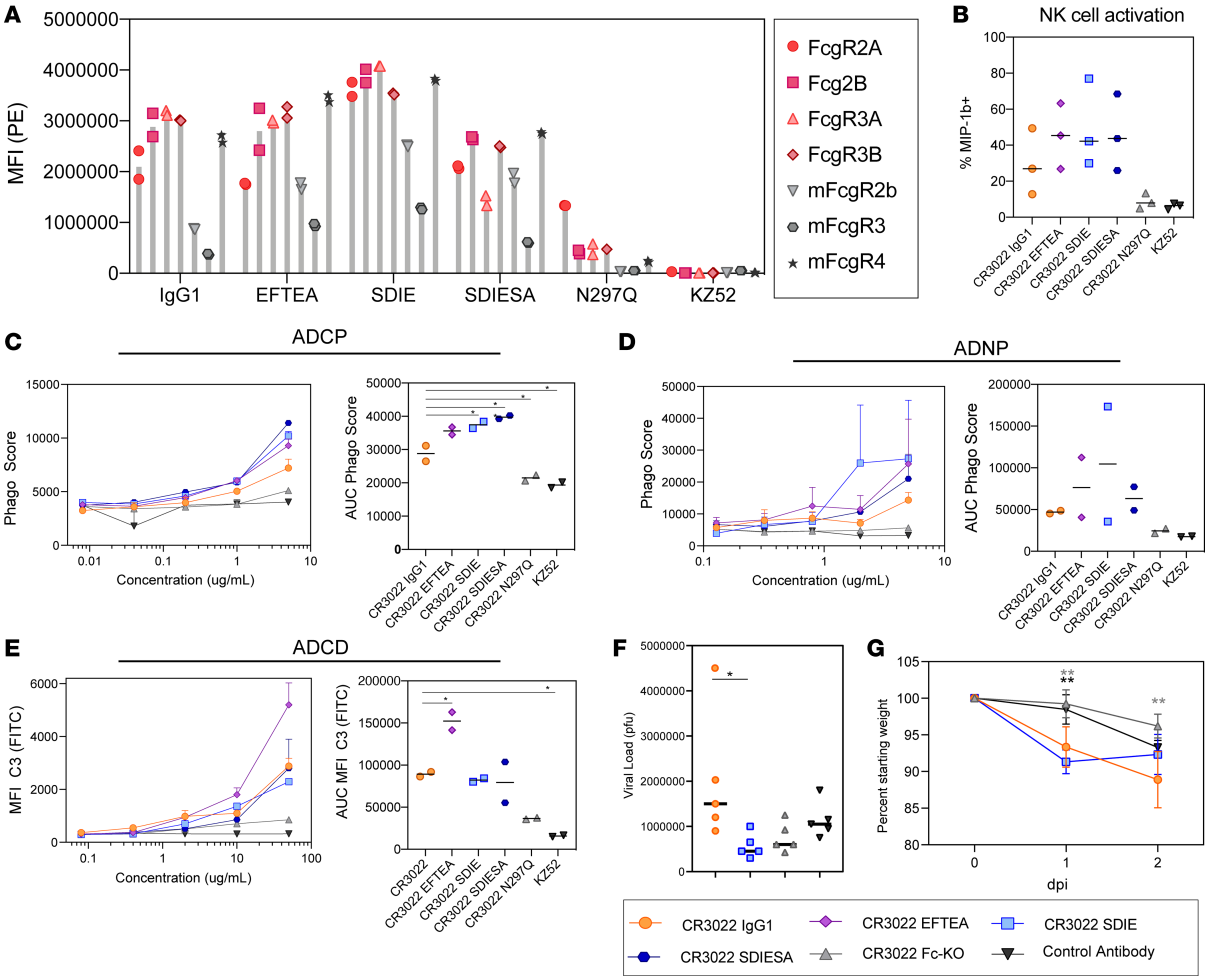
CR3022 can be enhanced through Fc engineering. (**A**) Each CR3022 variant was analyzed for its ability to bind the human FcγR2a, FcγR2b, FcγR3a, and FcγR3b or the mouse FcγR2b, FcγR3, and FcγR4 by Luminex. Each bar represents the average MFI and error bars show standard deviation. (**B**) The CR3022 variants were analyzed for their ability to drive NK activation, as measured by MIP-1b activity. The bar graphs represent the average of 3 donors, and the error bars represent the standard deviation. For AUC, significance was determined by an ordinary 1-way ANOVA followed by Tukey’s multiple-comparison test. *P < 0.05. (**C**–**E**) The CR3022 variants were evaluated for their ADCP (**C**), ADNP (**D**), and ADCD (**E**) activity. The dots on the line graph represent the average of 2 replicates. The bar graphs represent the average AUC of 8 serial dilutions run as replicates, and the error bars represent the standard deviation. For AUC, significance was determined by an ordinary 1-way ANOVA followed by Tukey’s multiple-comparison test. *P < 0.05. (**F** and **G**) BALB/c mice (5 per group) were treated with a CR3022 variant or control therapeutically. Lung viral titers were determined 2 days postinfection (dpi) (**F**), and weight was monitored daily (**G**). For viral titer (**F**), significance was determined by an ordinary 1-way ANOVA followed by Tukey’s multiple-comparison test. *P < 0.05. For weight (**G**), significance was determined by an ordinary 1-way ANOVA followed by Tukey’s multiple-comparison test. *P < 0.05, **P < 0.01, between the respective antibody, indicated by the color of the asterisk, and the CR3022 WT. For weight (**G**), each dot represents a mouse, and the error bars represent the standard deviation.

Although Fc effector functions have been linked to protection from MERS infection in mice (13), data have also emerged pointing to the potentially deleterious role of Fc effector functions in enhancing SARS-CoV disease (19, 29, 30). Thus, using a simple Fc-engineering approach where the variable domains of CR3022 are swapped onto the Fc domain of distinct Fc variants with previously defined point mutations known to alter antibody interactions with Fc receptors, we generated a small panel of CR3022 Fc variants, able to selectively augment phagocytosis, NK cell function, and complement activity. Given clear overlap and concordance in human IgG1 performance across human and mouse effector functions (31), we focused on known mutations in human IgG1 and compared human and mouse Fc receptor binding as well as human antibody effector functions. These mutations in the CH2 or CH3 of the Fc of IgG1 were previously identified to alter binding to FcγRs, resulting in enhancement or reduction of Fc effector function. Specifically, 4 mutants were explored: a mutant able to enhance all Fc effector functions (EFTEA, ref. 32), 2 mutations known to enhance ADCC and ADCP (SDIE and SDIESA, ref. 33), and a mutation that knocks out all Fc function (N297Q, ref. 34). To begin characterizing the function of these variants, we analyzed the ability of these mutants to bind both human FcγRs and mouse Fcγ receptors (mFcγRs) (Figure 3A). EFTEA showed similar binding to the human FcγRs as WT CR3022. SDIE exhibited slightly higher binding to the human FcγRs compared with WT, whereas SDIESA had a slight and selective reduction in binding to the human FcγR3A and FcγR3B. Conversely, the Fc-knockout mutation, N297Q, resulted in a near-complete loss of Fc receptor binding.

Although the variants bound the mouse FcγRs with lower affinity than the human FcγRs, most variants retained considerable binding to mFcγR4, an activating receptor in mice that has been implicated in antibody effector function (35). In particular, SDIE exhibited the highest binding to the mouse FcγRs compared with all other variants. Consistent with FcγR binding profiles, the EFTEA mutant exhibited enhanced pan-functionality, with a selective increase in ADCD (Figure 3E). Conversely, both SDIE and SDIESA exhibited enhanced ADCP and ADNP but not ADCD activity, with SDIE exhibiting enhanced ADNP compared with the other mutants (Figure 3, C–E). Finally, SDIESA exhibited the highest ADCP activity and NK activation, as measured by MIP-1b expression, of the group (Figure 3, B and C), highlighting the distinct functional profiles of each of the modifications. Although some residual binding was noted for N297Q on FcγR2A (Figure 3A), near-complete loss of antibody function was observed with this Fc variant (Figure 3, B–E), driving effector function at a similar level as the EBOV-specific control antibody, underscoring the dominant silencing effect of this mutation. Overall, these data highlight a range of functional variation across the Fc variants, enabling the down-selection of 3 variants for in vivo analysis: WT, Fc-knockout (Fc-KO), and pan-functional SDIE variant, which exhibited the highest binding to mouse FcγRs (Figure 3A).

### Dissecting therapeutic Fc signals of protection.

Using these down-selected Fc variants, we next aimed to probe the role of Fc effector function in protection from infection and disease (36). BALB/c mice were treated 12 hours following SARS-CoV-2 infection to probe for therapeutic protective Fc functions. Specifically, BALB/c mice were infected with 10^5^ PFU of mouse-adapted SARS-CoV-2 i.n.; treated with 200 μg of the WT, Fc-KO, or control antibody or 100 μg of the Fc-enhanced antibody i.p.; and monitored for 2 days for lung viral titer and weight loss, with 5 mice per group (Figure 3, F and G).

Strikingly, slightly higher, but nonsignificant, differences were observed in viral replication using the WT CR3022 IgG1 compared with the control antibody and the WT antibody (Figure 3G). Moreover, reduction in viral load was observed with the Fc-enhanced CR3022 variant (Figure 3G). However, significant weight loss occurred in both WT and Fc-enhanced variant-treated mice and minimal to no weight loss in mice treated with the Fc-KO variants (Figure 3G). Thus, while the WT CR3022 IgG1 and the Fc-enhanced CR3022 variants showed divergent virologic effects, both variants led to enhanced pathology. The disconnect between viral load and weight loss for the Fc-enhanced CR3022 antibody raised the possibility that the Fc-enhanced variant may have pathological consequences.

**Figure 4:**
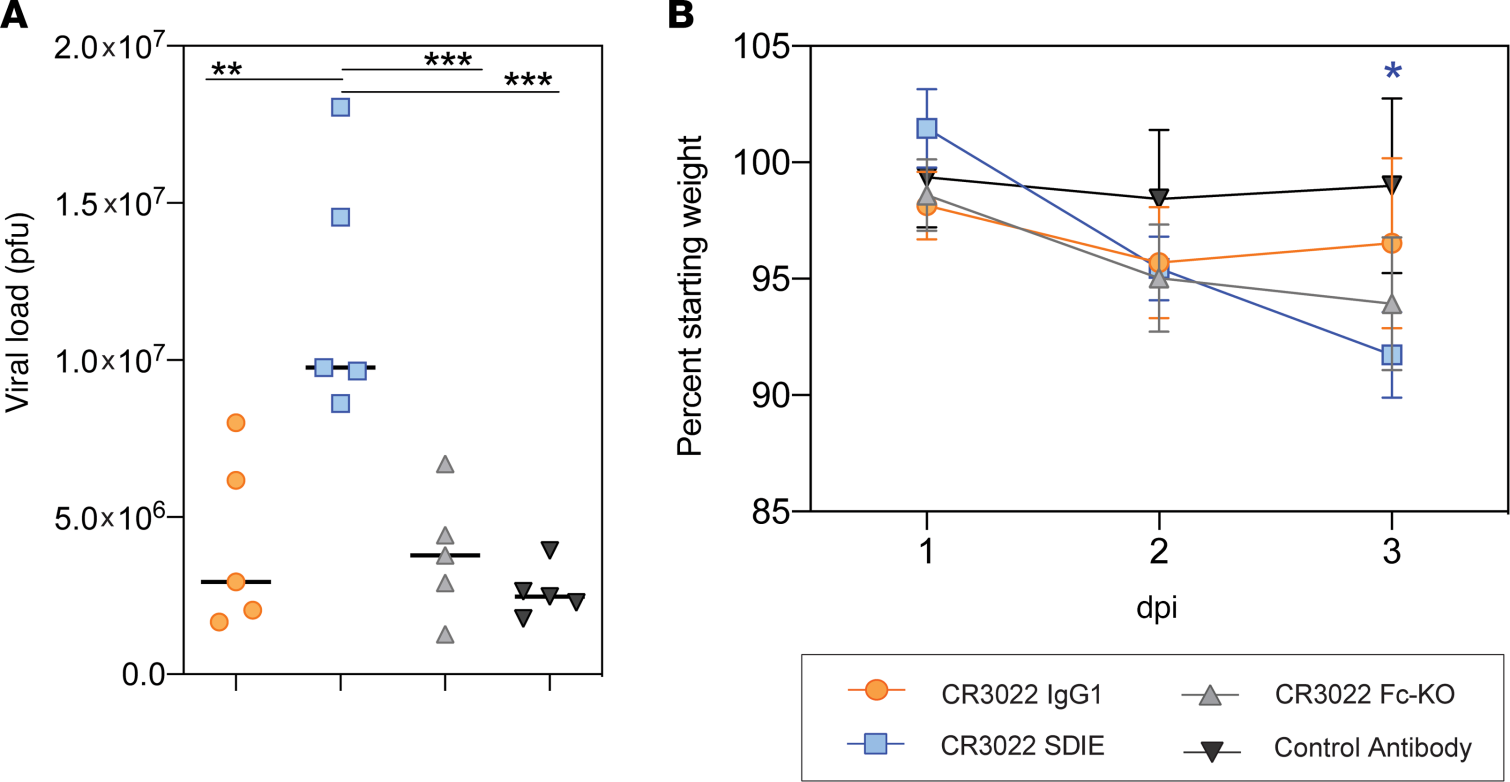
Increased Fc function causes enhancement of disease in vivo. Hamsters (5 per group) were challenged with SARS-CoV-2 and treated with a CR3022 variant or control antibody 1 dpi. Lung viral titers were determined 3 dpi (**A**), and weight was monitored daily (**B**). For viral titers (**A**), significance was determined by an ordinary 1-way ANOVA followed by Tukey’s multiple-comparison test. **P < 0.01, ***P < 0.001. For weight (**B**), significance was determined by 2-way ANOVA test followed by Tukey’s multiple-comparison test. *P < 0.05 between the respective antibody and the CR3022 WT. For weight (**B**), each dot represents the average of 5 hamsters, and the error bars represent the standard deviation.

To further understand the role of Fc-enhanced pathology, we next tested the therapeutic benefit of the CR3022 variants in a more pathological model of SARS-CoV-2 infection (37, 38). Syrian golden hamsters are highly susceptible to SARS-CoV-2 infection and develop severe infection after challenge. Thus, using this model, we assessed the panel of Fc-engineered monoclonals. Hamsters were challenged i.n. with 10^6^ PFU/mL (10^7^ TCID_50_) of SARS-CoV-2, and 1 day after infection, treated with 5 mg/kg IgG1, Fc-enhanced, Fc-KO, or a control antibody, with 5 hamsters per group. Weight was monitored daily, and lung viral titers were determined 3 days after infection (Figure 4, A and B). Similar to the results observed in the mouse model (Figure 3G), the WT CR3022 had no impact on viral load compared with control antibody–treated animals (Figure 4A). In contrast to mice, hamsters did not experience any benefit from the Fc-KO antibody (Figure 4A), likely due to the more severe nature of the infection in this model. Conversely, hamsters treated with the Fc-enhanced CR3022 exhibited increased viral load in the lung and increased weight loss (Figure 4, A and B). Despite the viral load disparity across the mice and hamsters, both models exhibited increased weight loss upon treatment with the Fc-enhanced CR3022 (Figure 3G and Figure 4B), suggesting similar host responses to the Fc-enhanced monoclonal. Thus, collectively, these data point to the critical importance of balancing Fc effector function to temper pathology in susceptible populations.

## Discussion

Given the rapid spread of SARS-CoV-2, therapeutics are urgently needed to not only prevent but also treat COVID-19. Among the strategies, passive transfer of monoclonal antibodies, which are able to both drive directed antiviral activity and also tune the immune system, represent an ideal class of therapeutics, potentially suited for both prevention and therapy. However, emerging data pointing to the possibility of antibody enhancement of disease following vaccination against SARS-CoV have raised the importance of carefully considering the role of the antibody Fc in SARS-CoV-2 therapeutic design. Here we focused on a first-in-class cross-SARS monoclonal antibody, CR3022, which interacts with a conserved region of the RBD. Although the antibody itself was highly functional, even in the presence of ACE2 (Figure 1), additional Fc engineering was performed, allowing us to gauge the therapeutic benefits of Fc activity (Figure 3). Surprisingly, the Fc-enhanced CR3022 antibody conferred some viral control when administered in mice but was accompanied by significant morbidity in both mice and hamsters, potentially by promoting an inflammatory response.

Past reports have shown some evidence of antibody-dependent enhancement (ADE) of SARS infection. Although many of these studies rely on in vitro systems with high levels of virus, diluted serum, or nonfunctional antibodies (39, 40), a more recent study in nonhuman primates pointed to a disease-enhancing role of modified vaccinia Ankara-induced SARS-CoV neutralizing antibodies (19). In vitro data suggested that despite the neutralizing activity of the vaccine-induced antibodies, ADE in SARS infection was caused by FcγR2-mediated activation of myeloid cells (41, 42). ADE is most well documented during Dengue virus infection, in which previous infection places an individual at risk, upon reinfection, to develop Dengue hemorrhagic fever/Dengue shock syndrome (DHF/DSS). This disease enhancement is thought to occur due to the presence of antibodies directed at a different Dengue serotype. Specifically, subneutralizing levels of preexisting antibodies to one serotype that have limited cross-reactive neutralizing capacity to a second serotype are enriched in children who develop DHF/DSS following exposure to a new serotype of the virus (43). Moreover, in vitro, low levels of neutralizing antibodies have been shown to facilitate viral entry into myeloid cells following FcγR engagement, resulting in enhanced infection and consequent inflammation (44). However, it is critical to note that limited evidence exists for SARS-CoV-2 infection of myeloid cells (45, 46). Instead, SARS-CoV-2 target cells do not express FcγR, and thus enhanced infection is unlikely to occur in the same manner as for Dengue viral infection. Conversely, SARS-CoV-2 target cells do express the neonatal Fc receptor, FcRn (47, 48). Whether enhancement can be caused by FcRn remains unclear. Yet, enhanced FcγR engagement may deliver virus preferentially to endosomal compartments in phagocytes, resulting in viral sensing and inflammatory responses, that may then lead to inflammation, cellular recruitment, and potential pathology. Thus, antibodies in SARS-CoV-2, unlike Dengue viral infection, may cause enhanced inflammation rather than enhanced infection. Importantly, here both FcγR-engaging antibodies drove pathology in both mice and hamsters but showed differences in viral loads. These data point to a disconnect between viral load and pathogenesis that may be dissected in the future with in-depth immunohistopathological studies across the 2 animal models.

It is critical to note that antibodies elicited by infection and vaccine platforms or monoclonal antibodies do not have the same Fc binding profiles as those induced by modified vaccinia Ankara (MVA) vaccination that was previously associated with SARS-CoV–enhanced disease in macaques (49). Importantly, distinct Fc binding profiles can be generated following infection and vaccination, driven by altered Fc subclass selection and Fc glycosylation (49). Moreover, previous studies have clearly illustrated striking differences in antibody functional profiles across MVA-, pox virus–, adenovirus-, and protein-based immunization strategies (50). Thus, it is plausible that polyclonal pools of antibodies, with neutralizing properties and balancing Fc receptor binding profiles via balanced Fc glycosylation, may provide protection in the absence of disease. Along these lines, recent vaccine studies point to a positive predictive role of polyclonal Fc recruiting directed at both the whole spike and RBD of SARS-CoV-2 (28, 51). Similarly recent monoclonal therapeutic studies with neutralizing WT IgG1 demonstrate limited evidence of disease in humans (52).

Despite the significant loss of most Fc effector functions in the Fc-KO variant (Figure 3), this variant retained low-level binding to human FcγR2A, involved in phagocytosis in humans (53). This remaining Fc receptor binding may have contributed to low but sufficient levels of immune complex–based activation in the susceptible hamster model, where even the Fc-KO variant drove weight loss. These data point to the ultrasensitive nature of the hamster model. Although mice exhibit more attenuated disease, hamsters suffer highly pathological responses to SARS-CoV-2 (37). Whereas the Fc-KO exhibited reduced viral and no weight loss in the mice, the administration of the Fc-enhanced variant resulted in pathology. Conversely, the more susceptible hamster model showed no benefit with any of the Fc variants and instead exhibited the same enhanced pathology with the Fc-enhanced. Thus, Fc enhancement may represent a liability across the disease spectrum. However, it is critical to note that many distinct Fc modifications can be utilized to drive enhanced biological activity. Although the SDIE mutation used here improved all measured Fc effector functions, other mutations exist that selectively improve NK cell activity, monocyte phagocytosis, neutrophil activation, or complement deposition, offering potentially more precise mechanisms to control infection. Moreover, recent vaccine correlates analysis in nonhuman primates highlighted the complementary activity of monocyte phagocytosis and complement in viral control (28, 51). Whether these functions alone may selectively clear the virus and prevent inflammation and pathology remains unclear but could provide critical clues for the strategic engineering of monoclonal antibodies to maximize protection and minimize pathology.

As the number of COVID-19 cases rise globally, new therapies are urgently needed to treat this highly infectious virus. Here, we characterized the therapeutic functions of the monoclonal antibody CR3022 that binds to a conserved site on the RBD that is not fully blocked in the presence of ACE2, offering therapeutic benefit even after infection has been initiated. The data presented here show no effect of CR3022 as a WT IgG1. Surprisingly, both the Fc-enhanced and Fc-silenced variants of CR3022 showed an antiviral benefit in mice but resulted in dichotomous treatment-associated pathology. Interestingly, the same pathological phenotype was observed in hamsters with the Fc-enhanced variant, highlighting the consistent disease-enhancing phenotype of highly functional non-neutralizing monoclonal variants. With the rapid discovery of novel neutralizing antibodies and pan–cross-reactive CoV antibodies, coupled to rapid Fc engineering, enabling the potential to deeply profile the involvement of all Fc receptors (including FcγR1, FcγR2a, FcγR2b, FcγR3a, FcγR3b, FcRn, as well as noncanonical Fc receptors) and the generation of functionally optimized antibodies with appropriate half-lives, the development of therapeutics with the highest clinical benefit is possible.

## Methods

### Cell lines.

THP-1 cells, originally isolated from a 1-year-old male human (ATCC), were maintained in RPMI supplemented with 10% fetal bovine serum, l-glutamine, HEPES, penicillin/streptomycin, and 0.01% β-mercaptoethanol. Vero E6 cells, from BEI Resources, National Institute of Allergy and Infectious Diseases (NIAID) NIH: VERO C1008 (E6), African green monkey kidney, Working Bank NR-596, were maintained in humidified incubators at 37°C and 5% CO_2_ in Dulbecco’s modified Eagle medium (DMEM) with GlutaMAX and sodium pyruvate supplemented with 10% (*v/v*) certified US-origin heat-inactivated fetal bovine serum (HI-FBS).

### Viruses.

SARS-CoV-2 USA-WA1/2020 (54) was propagated on Vero E6 cells in DMEM supplemented with 2% HI-FBS, GlutaMAX, sodium pyruvate, nonessential amino acids, and antibiotic-antimycotic. At 62 hours, the supernatant was harvested and clarified by centrifugation. The final concentration of HI-FBS was diluted to 10% (*v/v*) prior to cryopreservation at –80°C. Final passage was VERO+3, Vero E6+2 (lot NSU-V004). The sequence of this stock was identical to the published reference consensus sequence (54).

For in vivo studies, a recombinant SARS-CoV-2 mouse-adapted variant was constructed by introduction of 2 amino acid changes in the ACE2 binding pocket. Virus stocks were grown on Vero cells and titered by plaque assay as previously described by our group (36).

### Animals.

Female 12-month-old BALB/c mice were obtained from Envigo (strain 047). All animal work was approved by Institutional Animal Care and Use Committee at University of North Carolina at Chapel Hill. Syrian golden hamsters, 6–7 weeks old, were from and maintained at University of Texas Medical Branch.

### Plasmid design.

To create the CR3022 variants, gene blocks were designed containing the Fc domain of IgG1 and previously defined, individual Fc point-mutant backbones with known differences in binding to Fc receptors and functional differences (32–34). These Fc domains were cloned into individual pUC donor plasmids. In addition, 3 pUC plasmids encoding the variable heavy chain, a furin P2A sequence, or the variable light chain were designed surrounded by BsaI sites. In addition to the 4 pUC plasmids, a destination vector was cloned with an IL-2 secretion signal, the suicide gene ccdB surrounded by BsaI sites, and the kappa light chain sequence. The 4 pUC donor plasmids and the destination vector were combined in a single digestion-ligation reaction, using Golden Gate cloning, to create full IgG molecules with the same CR3022 antigen-binding (Fab) domain but different Fc domains.

### Protein expression and purification.

The RBD (residues 319–529) of SARS-CoV-2 S protein (GenBank: MN975262.1) were subcloned into a pVRC vector with a C-terminal SBP-tag.

The CR3022 (GenBank: DQ168569 and DQ168570), B38 (27), and 0012C10 and 0012E4 antibodies (provided in-house) were produced in 293F suspension cells grown in FreeStyle 293 Expression media (Gibco, Thermo Fisher Scientific). Cells were transfected with Polyethylenimine (PEI; Polysciences) at 1 μg/μL in a ratio of 3 μg PEI to 1 μg DNA. Supernatants were harvested 5 days posttransfection, and antibody was purified using protein G magnetic beads (MilliporeSigma). For in vitro analysis, KZ52 (Mayflower Bioscience, 0260-001) was used as a negative control.

### ELISA.

ELISA plates were coated with 50 ng/well of antigen in PBS overnight at 4°C on a shaker at low speed. The next day, plates were washed 5 times with PBS-0.05% Tween-20 (PBST) and blocked in 5% BSA in PBS for 2 hours at room temperature on a shaker at low speed. Plates were washed 5 times with PBST, and 5-fold serially diluted antibody was added and incubated for 2 hours at room temperature on a shaker at low speed. After the incubation, plates were washed with PBST and anti–human IgG1–HRP was added for detection. Plates were incubated for 1 hour at room temperature on a shaker at low speed. Plates were washed with PBST. The ELISA was developed with the addition of TMB (Invitrogen, Thermo Fisher Scientific). The reaction was stopped with 1 M H_2_SO_4_. Signal reading was carried out at 450 nm (reference wavelength of 570). Data were reference value and background corrected.

### ADCP assay.

The ADCP assay was adapted from Ackerman et al. (55). Briefly, antigen was biotinylated using sulfo-NHS (N-hydroxysulfosuccinimide, Pierce, Thermo Fisher Scientific, A39269) LC-LC biotin, coupled to yellow-green fluorescent Neutravidin 1 μm beads (Invitrogen, Thermo Fisher Scientific, F8776) for 2 hours at 37°C and washed 3 times in 0.1% BSA in PBS. The coupled beads were resuspended to a final volume of 10 μg/mL. A total of 10 μL/well of coupled beads were added to 96-well plates with 100 μL/well of antibodies at a concentration of 5 μg/mL, 1 μg/mL, 0.2 μg/mL, and 0.04 μg/mL for 2 hours at 37°C to form immune complexes. After incubation, the immune complexes were spun down and the supernatant was removed. THP-1 cells (ATCC) were added at a concentration of 2.5 × 10^4^ cells/well and incubated for 18 hours at 37°C. After incubation, the plates were spun down, the supernatant was removed, and cells were fixed with 4% PFA for 20 minutes. Fluorescence was acquired with an Intellicyt iQue. Phagocytic score was calculated using the following formula: (percentage of FITC^+^ cells × the geometric MFI of the FITC^+^ cells)/10,000.

### ADNP assay.

The ADNP assay was adapted from Karsten et al. (56). Antigens were coupled to beads and immune complexes were formed as described for ADCP. Neutrophils were isolated from freshly drawn whole blood. Erythrocytes were lysed with ammonium-chloride potassium lysis buffer (150 mM NH_4_Cl, 10 mM KHCO_3_, 0.1 mM Na_2_ EDTA, pH 7.4), and leukocytes were separated out by centrifugation, 500*g* for 5 minutes at room temperature. Leukocytes were washed with cold PBS, resuspended in R10, and added to plates at a concentration of 5 × 10^4^ cells/well. The plates were incubated for 1 hour at 37°C. The neutrophil marker CD66b (Pacific Blue–conjugated anti-CD66b; BioLegend, 305112) was used to stain cells. Cells were fixed for 20 minutes in 4% paraformaldehyde (PFA). Fluorescence was acquired with an Intellicyt iQue, and the phagocytic score was calculated as described for ADCP.

### ADCD assay.

The ADCD assay was adapted from Fischinger et al. (57). Antigen was coupled to red fluorescent Neutravidin 1 μm beads (Invitrogen, Thermo Fisher Scientific, F8775) as described for ADCP. Immune complexes were formed by incubating 10 μL of coupled beads with 50 μL of antibody at concentrations of 50 μg/mL, 10 μg/mL, 2 μg/mL, and 0.4 μg/mL for 2 hours at 37°C. Plated were spun down, and immune complexes were washed with PBS. Lyophilized guinea pig complement (Cedarlane, CL4051) was resuspended in 1 mL of cold water, diluted 1:50 in GVB++ (gelatin veronal buffer and additional Ca2^+^ and Mg2^+^, Boston BioProducts, IBB-300X), and added to the immune complexes. The plates were incubated for 20 minutes at 37°C, and the reaction was stopped by washing the plates twice with 15 mM EDTA in PBS. To detect complement deposition, plates were incubated with fluorescein-conjugated goat anti–guinea pig complement C3 (MP Biomedicals, 0855385) for 15 minutes in the dark. Fluorescence was acquired with an Intellicyt iQue.

### ADNKA (NK activation).

Human NK cells were isolated from buffy coats using RosetteSep NK cell enrichment kit (StemCell Technologies) and Ficoll separation. The isolated NK cells were rested overnight at 1.5 × 10^6^ cells/mL in IL-15 at 37°C. ELISA plates were coated with antigen at 300 ng/well and incubated for 2 hours at 37°C. Plates were blocked with 5% BSA in PBS overnight at 4°C. The next day, 100 μL of antibodies, at a concentration of 5 μg/mL, were added to the plates. Plates were incubated for 2 hours at 37°C to form immune complexes. After the incubation, NK cells were added to the plates at 5 × 10^4^ cells/well in R10 supplemented with anti-CD107a PE-Cy5, Brefeldin A (MilliporeSigma, B7651-5MG), and GolgiStop (BD Biosciences, 555802). Plates were incubated for 5 hours at 37°C. Following the incubation, NK cells were stained for the surface markers with anti-CD56 PE-Cy7, anti-CD16 APC-Cy7, and anti-CD3 Pacific Blue (BD Biosciences, 557747, 557758, 558124). NK cells were fixed and permeabilized with Fix&Perm cell permeabilization kit (Invitrogen, Thermo Fisher Scientific). Cells were incubated with anti–MIP-1β PE and anti–IFN-γ FITC (BD Biosciences, 550078, 340449) to stain for intracellular markers. Cells were acquired on an Intellicyt iQue.

### FcR binding.

A multiplex assay was used to determine FcR binding as described in Brown et al. (58, 59). A 2-step carbodiimide reaction was used to couple antigen to Magplex Luminex beads. Beads were activated for 30 minutes at room temperature using 100 mM monobasic sodium phosphate, pH 6.2, with 5 mg/mL sulfo-NHS and 5 mg/mL ethyl dimethylaminopropyl carbodiimide hydrochloride. Beads were then washed with 50 mM 2-(N-Morpholino)ethanesulfonic acid (MES), pH 5.0, and incubated with 25 μg of antigen in 50 mM MES, pH 5.0, for 2 hours on a rotator. The coupled beads were blocked in Blocking Buffer (PBS, 0.1% BSA, 0.02% Tween-20, 0.05% Azide, pH 7.4). After blocking, coupled beads were washed in PBS-Tween, resuspended in PBS, and stored at 4°C.

For the detection of FcR binding, FcRs with an AviTag were biotinylated using a BirA500 kit (Avidity) per the manufacturer’s instructions. Coupled beads were diluted to a concentration of 100 microspheres per antigen/μL in 0.1% BSA in PBS. Antibodies were serially diluted in 0.1% BSA in PBS; mixed with diluted beads in a black, clear-bottom, 384-well plate; and incubated at 4°C for 16 hours, shaking at 900 rpm. After the incubation, plates were washed with 0.1% BSA in PBS. FcRs were incubated with streptavidin-PE (Prozyme, PJ31S) for 10 minutes. PE-labeled FcRs were added to plates and incubated for 1 hour at room temperature on a shaker. Plates were washed with 0.1% BSA in PBS and resuspended in Qsol Buffer (Intellicyt). Fluorescence was acquired on the Intellicyt iQue.

### In vitro plaque reduction neutralization assay.

The day prior to assay, VeroE6 cells were seeded to a density of 8 × 10^5^ cells/well in 6-well plates. SARS-CoV-2 was diluted in DMEM with GlutaMAX and sodium pyruvate supplemented with 1× antibiotic-antimycotic and 2% HI-FBS to 1000 PFU/mL (target 100 PFU per well). Antibody was serially diluted in Dulbecco’s PBS, and an equal volume of diluted SARS-CoV-2 was added, mixed, and incubated for 1 hour at 37°C before plating on 6-well plates (200 μL in triplicate). Following a 1-hour incubation at 37°C with periodic rocking, they were overlaid with a 1:1 mixture of 2.5% (*w/v*) Avicel RC-591 (provided by DuPont Nutrition & Health) prepared in distilled water and 2× Temin’s Modified Eagle Medium (Thermo Fisher Scientific) supplemented with 10% HI-FBS, 2× GlutaMAX, and 2× antibiotic-antimycotic. Following a 2-day incubation at 37°C and 5% CO_2_, plates were fixed with 10% neutral buffered formalin for removal from biocontainment and stained with a solution of 0.2% Gentian Violet and 10% neutral buffered formalin. Plates were rinsed under water and plaques were enumerated. Percentage neutralization was calculated from vehicle/virus-only control wells.

### In vivo challenge.

Female 12-month-old BALB/c mice were treated prophylactically (12 hours before infection) or therapeutically (12 hours after infection) with 200 μg or 100 μg of antibody through the intraperitoneal route. Each group contained 5 mice. Mice were challenged i.n. with 10^5^ PFU of mouse-adapted SARS-CoV-2, representing 0.69 × 10^5^ TCID_50_, which falls clearly in the range of viral loads observed in hospitalized patients (60). Weight was monitored on days 0, 1, and 2 after infection. Mice were sacrificed 2 days after infection, and lung viral titer was determined by plaque assay. Although the Fc variants experience different half-lives in vivo, the studies performed here were short; but half-life should be considered for longer studies.

Adult hamsters were microchipped a day prior to experimental challenge. On day 0, hamsters were anesthetized with ketamine/xylazine and challenged with SARS-CoV-2 by the i.n. route using a 10^7^ TCID_50_ (or 10^6^ PFU/mL) dose in a total volume of 100 μL. The final challenge dose was 10^4^ PFU diluted in sterile PBS. Body weight and body temperature were measured each day, starting at day 0. On day 1 postchallenge (1 dpc) hamsters were treated with 5 mg/kg of monoclonal antibodies diluted in 0.5 mL of sterile PBS via the intraperitoneal (i.p.) route. The control group got an equal volume of sterile PBS via the same i.p. route. On 3 dpc all animals were sacrificed. At necropsy, lungs were harvested for all groups. Left lungs were fixed with 10 volumes of fresh 10% formalin; right lungs were frozen in 5 mL lysogeny broth from Thermo Fisher Scientific for viral load analysis. Tissue sections were homogenized in bead beater tubes (Thomas Scientific) and weighed, and supernatants were titrated per standard protocol. Briefly, 100 μL of a 10× dilution of supernatants was incubated in 96-well plates for 1 hour, and supernatants were replaced by methyl cellulose overlay and incubated for 3 days at 5% CO_2_ and 37°C. Plates were fixed with formalin and removed from the biosafety level 4 facility, after which the plates were inactivated and immunostained, and the plaques were counted to obtain viral titers.

### Statistics.

All data were visualized and analyzed in GraphPad Prism. Nonparametric tests were performed as described in figure legends. Where applicable, significance was determined as *P < 0.05, **P < 0.01, ***P < 0.001.

### Study approval.

Primary human innate immune cells were isolated from fresh peripheral blood samples collected by the Massachusetts General Hospital (MGH) blood bank. All subjects provided informed consent, and the study was approved by the MGH Institutional Review Board. All subjects were older than 18 years of age, and samples were deidentified prior to use.

All mouse work was approved by the Institutional Animal Care and Use Committee at the University of North Carolina at Chapel Hill. The animal protocols for the hamster models were approved by the Institutional Animal Care and Use Committee of the University of Texas Medical Branch.

## Author contributions

CA, MDS, AG, TS, BDJ, JG, DRW, AB, RB, and GA designed the study. CA, SF, MDS, JB, AS, SRL, NAK, CM, AH, RJ, NS, PT, TZ, JL, AZ, and CL performed experiments. MB, DRW, and JRD provided reagents. CA performed all analysis.

## Figures and Tables

**Table 1:**
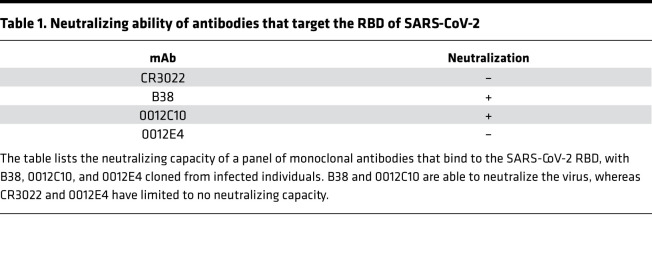
Neutralizing ability of antibodies that target the RBD of SARS-CoV-2

## Version 1 01/11/2021

Electronic publication
